# The translation process of the culturally sensitive active aging scale for community-dwelling older adults in Pakistan

**DOI:** 10.1186/s12889-023-16563-1

**Published:** 2023-08-31

**Authors:** Rashida Bibi, Zhang Yan, Akhter Zeb, Nasir Anwar, Nasar Mian, Roheeda Amanullah Khan

**Affiliations:** 1 Institution of Nursing and Health Sciences, Zhengzhou University, Zhengzhou, China; 2 Ismail Nursing College Sawat, Sawat, Pakistan; 3 National College of Nursing, Sawat, Khyber Pakhtunkhwa Pakistan; 4 Odhyana College of Nursing, Sawat, Khyber Pakhtunkhwa Pakistan; 5 Government Nursing College, Hayatabad Medical Complex, Peshawar, Pakistan

**Keywords:** Psychometric, Translation, Culturally sensitive, Active aging, Scale, Urdu, Older adults, Pakistan

## Abstract

**Background:**

The assessment of active aging levels in Pakistani older adults is crucial yet; research tools are scarce in the local language. Therefore, this study aims to translate and validate the English version of an Active Aging Scale into a cross-culturally sensitive Urdu version to assess active aging levels in Pakistani older adults.

**Methods:**

To translate and validate the scale, we used the ISPOR (International Society for Pharmacy Economic and Outcome Research) standards. Reliability, concurrent validity, construct validity, convergent validity, and discriminatory validity were checked on a total sample of 160 community-dwelling older adults. After two weeks, the test–retest reliability was examined. AMOS version 23 and SPSS version 23 were used to analyze the data.

**Results:**

The average content validity index for clarity was 0.91 and relevancy was 0.80. The total variance in the pilot study of all items secured > 0.3 variances except for two items scored < 0.30 that were omitted before the validity and reliability test. The remaining items explained 65.46% of the overall variation and had factor loadings ranging from 0.46 to 0.90 in the principal factor analysis (PFA). The confirmatory factor analysis of the Active Aging Scale revealed that the model fit was good with a Chi-square value (418.18 (DF = 2.2) which is less than 3.00. This is further evidenced by the root mean square error of approximation (RMSEA) of 0.042, goodness of fit index (GFI) of 0.92, adjusted goodness of fit index (AGFI) of 0.94, and comparative fit index (CFI) values of 0.92 and 0.96 (unstandardized and standardized, respectively). The scale’s Cronbach’s alpha coefficient was 0.88, indicating dependability and its test–retest reliability with the significance of (*P.* < 0.05).

**Conclusion:**

The Urdu version of the Active Aging Scale was successfully translated and validated in a culturally sensitive manner, and can be used to evaluate the effectiveness of various active aging interventions for older adults in Pakistan.

## Introduction

The older adult population in Pakistan has been increasing, resulting in public healthy challenges and poor quality of life for older adults living in the community [1]. According to the population census of 2017, there are over 25 million senior citizens in Pakistan, comprising approximately 6 to 10 percent of the total population [2, 3]. In addition, Pakistan is a Muslim country where predominantly its inhabitants are Muslims [1, 4]. It is concerning for researchers and policymakers that community-dwelling older adults in Pakistan face multiple challenges such as neglect within the healthcare system [2, 5–7], and a value system in which male family members are responsible for conducting activities outside home and females have fewer opportunities. Therefore females are at a higher risk to develop physical and mental health issues [8, 9], which lead to reduced social participation, self-care, productivity, and overall poor quality of life [10, 11]. However, studies highlighted the significant role older adults can play in society's development and financial stability if they receive support and encouragement [8, 12, 13]. Therefore, implementing strategies for active aging becomes crucial to enable older adults to spend healthy and independent lives.

To achieve this, culturally appropriate research instruments are needed to assess various aspects of older adults' active aging levels and promote active aging among older adults in Pakistan [14]. According to the previous studies, the research instruments should comply with conventional standards of validity and reliability, while also being simple and easily adaptable for older adults with low literacy [12], so the actual aspects of active aging can be evaluated [15].

Over the last two decades, different conceptual definitions, models, and measures related to aging, active aging, successful aging, and healthy aging emerged in the literature [16]. The World Health Organization's (WHO) policy framework [17], and life course program provide a comprehensive and widely recognized definition of active aging coincided with three pallor of active aging which involves enhancing health opportunities, societal participation, and safety and security when get old [11], to develop a worldwide healthy community. Active aging encompasses multiple determinants such as employment, social participation, empowerment, healthy lifestyle, participation in leisure activities, social contributions, and having financial security. The WHO policy framework emphasizes the importance of being healthy, participating in society, and financial security in old age to spend high quality of life [17].

Several scales have been developed by researchers in previous studies to assess status of active aging in older adults [18]. The Successful Aging Index (SAI) developed by Meredith Troutman, consists of four domains and 20 items [19], Kattika Thanakwang developed the Active Aging Scale (AAI) with seven factors and 36 items for older adults in Thailand [14]. Ziadi et al. developed a scale with four domains, including employment opportunities, social participation, independent and healthy living, and active aging capacity [20], Eun Lee developed the Active Aging Inventory (AAI) in the United States [21] incorporating four domains based on WHO's active aging strategies [21]. These scales are validated, and reliable in their respective cultural settings, encompassing various dimensions of active aging. All the scales were significantly correlated with measures such as the healthy aging scale, successful life scale, and empowerment scale [22, 23]. Culturally sensitive instruments are crucial to collect unbiased data, as directly adopting scales from different cultures may not fulfill the criteria of cross-cultural sensitivity [24]. While multiple scales exist in the literature to assess active aging in older populations in other countries. After a literature review, a scale developed by Kattika Thanakwang and colleagues, and translated into English by Tania Rantanant in Taiwan [15] was selected, which aligns with the WHO's proposed dimensions of active aging, and shows valid and reliable scale covered all aspects of active aging (Cronbach Alfa 0.92). This study tool can be translated into the Urdu language for community-based older adults in Pakistan. This scale consists of 36 items and a four-point rating scale available in English version. It has undergone translation processes in various languages to ensure cultural appropriateness. The translated instrument's construct validity needs to be confirmed, considering the diverse populations and distinct cultures in Western and other Asian civilizations where these scales have been utilized [22, 25]. It is worth noting that there is no universal agreement on the adaptation process of research scales [24]. These scales have undergone adaptation processes, ensuring validity and reliability in cross-cultural settings multiple times [26].Therefore, it is crucial to translate and develop culturally sensitive active aging scales to collect impartial data regarding active aging from older adults in Pakistan. The construct validity and relevance of the translated instrument need to be confirmed through rigorous testing in the local context [26]. By translating a cross-culturally relevant scale, researchers and policymakers will have a valuable tool to assess active aging in the geriatric population, contributing to future research and interventions aimed at promoting healthy aging in Pakistan. However, according to my knowledge, there is only one validated successful aging scale developed by Anwar et al. focused on only four domains of active aging such as life adaptation, and self-reliance [22, 26], which may not fulfill the holistic assessment of active aging. Therefore, there is a need to translate or develop a scale in the local context that can cover seven dimensions of active aging in Pakistani older adults. Therefore, this study aimed to translate and validate the active aging scale in the local language (Urdu) to assess active aging among community-based older adults in Pakistan.

## Materials and methods

### Phase.1

**Fig. 1:**
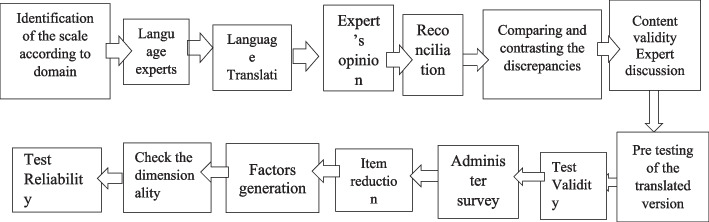
Flow chart of questionnaire translation& adaptation process

We followed the steps and recommendations made by the International Society of Pharmacy Economic and Outcome Research (ISPOR) report for the translation and cultural adaptation process [25], and the consensus-based standard for the selection of the COSMIN) checklist [27] was followed throughout the translation process of the scale (see Fig. 1).

#### Step.1 Selection of study instrument

An Active Aging scale was developed by Thannak Wang and others in Thailand [14] and translated by Tania Rantanen in English [15] was chosen to translate into Urdu language. After a literature review regarding the components of active aging, we approached the primary developer of the scale Thanakwang and secondary developer Rantanen through email. The active aging scale consisted of 36 items on five Likert scales from strongly agree = 5 points to strongly disagree = 1 point. The scale obtained Cronbach Alfa 0.92.

#### Step.2 Forward, transition

The scale was given to two independent bilingual translators to translate into Urdu language. During the translation process, there was no replacement or deletion of any items and maximum similarity of the source and target language was maintained.

#### Step.3 Expert comments to select the items

Two bilingual experts from Peshawar University and one Ph.D. scholar from the nursing department of Khyber Medical College were approached to select the best version for further process.

#### Step.4 Backward translation

Other two bilingual experts who were blind with the primary translators retranslated the translated version into the original language.

#### Step.5 Feedback study

Focus group of five experts (2 Ph.D. scholars in nursing and faculty members of Khyber Medical University, one community health nurse, a lecturer in government nursing college Peshawar, and one language expert professor Peshawar University and one general public figure) retired principal from the government were arranged to finalize the final draft on 22 March 2022. The committee evaluated each item, and they looked for agreement between the original scale and the translated English form. Assigning 1 to 4 scores for clarity and relevancy checked content validity index and scale validity. To communicate their greater insight, some of the words were placed within parentheses [28].

#### Step.6 Face validity

We followed a one-to-one approach to check the structure and its representativeness as a whole.

#### Step.7 Pilot testing

We conducted a pilot study on 30 participants to check the preliminary internal consistency of the scale. According to Yoshihito, et al. [23] at least 30 participants are required for a quantitative analysis of data, and to make decisions for further psychometric testing in a large scale. The participants in the pilot study were not included in the main study.

#### Step.8 Reliability of the pilot study data

Three criteria were used in the process of deciding which items to retain: 1) a minimum inter-item correlation of 0.20 and a maximum of 0.70, 2) a minimum corrected item–total correlation coefficient of 0.30, and 3) a minimum Cronbach’s reliability of 0.70 [14].

#### Ethical consideration

Before the collection of data, permission was obtained from the parent institution Zhengzhou University, Ethical Review Board, (ZZUIRB #202,254), and the District Health Department Office (DHO #14,207). We obtained verbal and written concerns from the participants.

### Phase.2

#### Study design and setting

To check the reliability and validity of the translated version of active aging scale, a cross-sectional study was conducted to collect data from 160 community based on older adults aged 60 years and above, from June 2022 to November 2022. The study was conducted in three districts of Khyber Pakhtunkhwa province in Pakistan such as district Peshawar, Timergara, and district Dir Lower.

#### Sampling method

A purposeful, convenient sampling method was followed to collect data from 160 community based older adults. The participants were representing a diverse cultural background such as Pashtun, Panjabi, Sindhi and northern area of Pakistan. The survey was conducted through translated Urdu version active aging scale to determine whether AAS-Pak could address the level of their active aging.

#### Inclusion and exclusion criteria

The following inclusion and exclusion criteria were followed: 1) Participants agreed to participate in the study. 2); not having a sensory or cognitive impairment. We excluded those who were having difficulties in understanding language, hospitalized older adults at the time of data collection, had disabilities due to any reason, or diagnosed with severe depression, and cognitive problem.

#### Sample size calculation

The sample size of this study was n = 160, calculated through G. Power sample size calculating software by keeping a desired level of significance(α = 0.05) with 95% of confidence level, and 20% of margin of error, considering 13% of the older population. According to MC Challun, 5 respondents for one item require for factor analysis of a scale [29].

### Data analysis

#### Reliability of the scale

For the general scale and its subscales, we measured reliability using Cronbach's alpha, and Pearson correlation coefficient values. For a new tool, a Cronbach's alpha value of 0.70 and higher suggests adequate internal consistency.

#### Ceiling and floor effect of the data

The proportion of respondents scoring the highest (ceiling) or lowest (floor) possible score across a given domain to assess the proportion of subjects scoring the best (maximum) or worst (minimum) score of each item. We considered the + 3 or -3 formula to examine the discrimination. Items below 15% of the threshold for missing item responses were removed.Items with the mean ± with standard deviation (SD) exceeding the range and with maximum and minimum scores exceeding 15% were deleted by applying the ceiling and floor effects methods used in the previous studies [30].

#### Validity of the scale

Exploratory factor analysis (EFA), confirmatory factor analysis, criterion validity, discriminatory validity, and structured validity were performed. In the EFA, established item weights (0.40) in principle component analysis to obtain a maximal reliable scale. KMO and the Bartlett test of Sphericity were applied to check the sample adequacy; factor matrices of loading for confirmatory factor analysis using methods of maximum likelihood factor analysis (MLFA) and principal component analysis (PCA) in the exploratory factor analysis model. Confirmatory CFA was employed with Amos version 23 to establish the construct validity of the instrument. We examined the magnitude of the loadings on each variable one by one and assessed the extent of variance accounted for. In the path analysis, components' relationships with assigned items in the model were assessed. Furthermore, in the verification of measurement error, we calculated the Chi-Square (χ^2^), comparative fit index (CFI) round-square standard error (RMSEA), goodness of fit index (GFI), the adjusted goodness of fit index (AGFI), and the relative fit index (RFI) in the model fit analysis. To check the structure validity of the instrument, different fit indices were applied to evaluate the overall fit model for the measures through structural equation modeling. Varimax with Kaiser Normalization rotation using least squares > o.4 an eigenvalue value > 1 value in factor loading [31]. All items with values less than 0.4 in Varimax rotation were deleted and the remaining items were reloaded for refactor analysis.

## Results

### Phase.1

#### Reliability of Pilot study

To check the reliability of the pilot study, we calculated Cronbach’s Alfa from 30 study participants. Results showed that the sum of item variance (S^2^Y) is 55.5324; while the variance of the total scores (S^2^) is 243.0916 on this translated scale. The Cronbach's alpha of the pilot study by applying the formula: 36/36–1*55.5324/243.0916–1.0.80. We know that Cronbach's alpha > 0.80 indicates that the scale is more reliable to apply in the study [32].

#### Content validity index

The average of I-CVIs is 0.93 for clarity and 0.88 for relevancy. If the I-CVI is higher than 83%, the item will be appropriate (see Table 1).

**Table 1:** CVI, SCVI of Clarity and Relevancy of the translated version of the Active aging scale

Comp	Items	TA	ICVI	UA	SCVI	TA	ICVI	UA	
Comp = 1	Scale Item 1		1	1	0.8889	5	0.833	0	0.967
	Scale Item 2	5	1	1		6	1	1	
	Scale Item 4	2	0.5	0		6	1	1	
	Scale Item 5	3	0.833333333	0		6	1	1	
	Scale Item 6	5	1	1		6	1	1	
	Scale item 7	5	1	1		6	1	1	
	Scale item 8	2	0.25	0		5	1	1	
	Scale item 9	5	1	1		6	1	1	
Comp = 2	Scale Item 10	5	1	1	0.9222	6	1	1	0.972
	Scale Item 11	5	1	1		5	0.833	0	
	Scale Item 12	5	0.833333333	1		6	1	1	
	Scale Item 13	4	1	1		6	1	1	
	Scale Item 14	6	1	1		6	1	1	
	Scale Item 15	3	0.50	0		6	1	1	
	Scale Item 16	6	1	1		6	1	1	
	Scale Item 17	5	1	1		6	1	1	
Component .3	Scale Item 18	4	0.666666667	1	O.93	6	1	1	0.96
	Scale Item 19	6	1	1		5	0.833	0	
	Scale Item 20	6	1	1		6	1	1	
	Scale Item 21	6	1	1		5	1	0	
	Scale Item 22	6	1	1		6	1	1	
Component.4	Scale Item 23	3	0.5	0	0.875	6	1	1	1
	Scale Item 24	6	1	1		6	1	1	
	Scale Item 25	5	1	1		6	1	1	
Component.5	Scale Item 26	5	0.833333333	1	0.9667	6	1	1	0.967
	Scale Item 27	6	1	1		5	0.833	0	
	Scale Item 28	6	1	1		6	1	1	
	Scale Item 29	6	1			6	1	1	
Comp 6	Scale Item 30	6	1	1	0.95	6	1	1	0.958
	Scale Item 31	6	1	1		6	1	1	
	Scale Item 32	6	1	1		5	0.833	0	
Comp7	Scale Item 33	6	1	1	1	6	1	1	1
	Scale Item 34	5	1	1		6	1	1	
**Total ICVI**		**0.933322**				**ICVI**	**0.96**		
**Total SCVI**		**0.949074**				**Total SCVI**	**0.97**		
**SCVI AVE**		**0.91666**				**SCVI AVE**	**0.88**		

### Phase.2

#### Demographic variables

The gender distribution was almost equal male 53.12% and female 46.87%, the interesting thing about this study is that nearly half of the participants, or 35%, were financially dependent on other family members. Majority (57.5%) of the participants' ages ranged from 66 to 78 years in this study. The education level among this group was very low, as 58.12% could only understand Urdu and had no formal schooling; more than that, only 1.25% were at the graduate level. Almost all (90.62%) of the participants live with their offspring or other family members, and only 9.37% reported living alone in this study. The burden of chronic diseases is very high among study participants as 58% reported at least one chronic condition (see Table 2).

**Table 2:** Demographic distribution of the study participants. *N* = 160

Variables	*N* = 160	Percent (%)
Age		
60 to 65 yrs	36	22.5%
66 to 70 yrs	92	57.5%
71 to 75 yrs	25	15.62%
> 76 yrs	7	4.37%
Marital status		
Married	135	84.37%
Widow	25	15.62%
Gender		
male	85	53.12%
female	75	46.87%
Living status		
alone	15	9.375%
with other families	145	90.625%
Health status		
One chronic disease	92	57.5%
> 1 chronic disease	39	24.37%
No chronic diseases	29	18.125%
Source of Income		
Pension	45	28.125%
Other	58	36.25%
Dependent	57	35.625%
Education status		
Illiterate	93	58.125%
Primary	45	28.125%
secondary	15	9.375%
high secondary	5	3.125%
Graduation	2	1.25%

#### Item, inter-item correlation

The item–total correlation, inter-item correlation, and inter subscale correlation of the 34-item were examined. The inter-subscale correlations ranged from 0.38 to 0.88. The reliability for the entire scale was 0.88, which is regarded as excellent, and the alpha coefficients for the seven subscales varied from 0.86 to 0.90 in this scale.

In the Table 3, the overall Cronbach Alpha is 0.88, and the 7 subscales ranged from 0.862 to 0.90, indicating good internal consistency; Pearson correlation among each variable is significant with *(P.0.001), and (P.* < *0.05*). *P*. value of SR, IS, HLT, SFT = (*P*. < 0.01), ES, SC, SW = (*P*. < 0.05).

**Table 3:** The correlation coefficient of inter subscale, subscale, and total scale and Alpha coefficient of the final draft of 34 items AAS-Pak

Table 3 Total scale components	SR	LIS	HLT	SW	ES	SFT	SC	Cronbach Alpha=^α^
Being self-reliance	1							0.87
Learning, integrated into society	0.490^**^	1						0.86
Healthy lifestyle	0.528^**^	0.478^**^	1					0.88
Developing spiritual wisdom	0.676^**^	0.409^**^	0.868^**^	1				0.89
Economic security	0.387^**^	0.405^**^	0.356^**^	0.388^**^	1			0.90
Strengthen family ties	0.676^**^	0.553^**^	0.418^**^	0.507^**^	0.688^**^	1		0.90
Contribution to society	0.393^**^	0.411^**^	0.478^**^	0.486^**^	0.384^**^	0.489^**^	1	0.88
Total scale correlation values	0.768^**^	0.845^**^	0.725^**^	0.709^**^	0.400^**^	0.006^*^	0.55^**^	1 0.81

*Abbreviations*: *SR* Self-reliance, *LIS* Learning and integrate into society, *HLT* healthy lifestyle, *SW* Spiritual wisdom, *ES* economic security, *SFT* Strengthen family ties, *SC* Social contribution

P*. < 0.005, P**. < 0.002,* P*.*88 < 0.001,

According to Gen, if the item-total score correlation coefficients are positive that is > 0.30, it means that the scale's items distinguish well by the people, provide examples of comparable behaviors, and have a high degree of internal consistency [33].

## Data adequacy

KMO and the Bartlett test of Sphericity ratio is > 0.5 for the data set to be considered suitable for further factor analysis [28]. The research revealed that the KMO coefficient was.0.701 (Table 4).

**Table 4:** KMO and Bartlett's Test

Kaiser–Meyer–Olkin Measure of Sampling Adequacy		.871
Bartlett's Test of Sphericity	Approx. Chi-Square	1999.463
	df	135
	Sig	0.000

### Findings regarding validity analysis results of AAS-Pak

#### Exploratory factor analysis

The findings showed that seven factors explained 65.4% of the total variance. All factor loadings had statistical significance and were higher than 0.40 in the Varimax rotation. Lastly, 34 elements were kept, and seven factors with eigenvalues greater than 1was formed. The eigenvalues ranged from 8.3 to 11.65, and the total variance was explained by all components in 65.48% of the cases. The factor loadings were statistically significant (0.001), and the commonality values ranged from 0.42 to 0.85, indicating that a large amount of the item variance was explained by the extracted components, organized theoretically, and had their best-defined structure (see Table 5).

**Table 5:** Factor* loading in principle factor analysis*

S#	**Items**	**Extraction**	**S#**	**Items**	**Extraction**
1	SR1	0.78	19	HL16	0.6
2	SR2	0.79	20	HL17	0.7
3	SR3	0.71	21	HL18	0.8
4	SR4	0.69	22	HL19	0.9
5	SR5	0.73	23	HL20	0.8
6	SR6	0.56	24	SC 21	0.5
7	SR7	0.43	25	SC21	0.7
8	ISL6	0.5	26	SC22	0.7
9	ISL7	0.7	27	ES23	0.9
10	ISL8	0.67	28	ES24	0.7
11	ISL9	0.67	29	ES25	0.42
12	ISL10	0.46	30	SW28	0.8
13	ISL11	0.82	31	SW26	0.7
14	ISL12	0.64	32	SW28	0.4
15	ISL13	0.38	34	SFT29	0.7
16	HL14	0.62	35	SFT30	0.42
18	HL15	0.68	36	SF 34	0.5

The component matrix in the factor analysis supported the seven components and their corresponding eigenvalues. The cross-loadings indicated relatively high loadings on more than one factor, and the item did not contribute to factor interpretability. The 34 retained items could all be meaningfully explained by only one of the seven factors and had loading values greater than 0.40 on all the retained items (see Table 5).

In Table 5 factor loading of 34 items in Active Aging-PAK, retained values range from 0.35 to 0.92 showing one dimension as > 0.40 except for one item which was accepted to be retained in principle factor analysis with Varimax rotations. According to Cigdem [28], factor load values are expected to be > 0.40 or higher. According to the factor load values, it has been observed that the scale consists of a diverse dimension and 34 items. Abbreviations are explained in Table 3 for reference.

#### Convergent validity

Convergent validity explained how much the indicators in each variable are close to each other. Convergent validity can be assessed by the average variance extracted (AVE) which referred to the degree the construct identifies the variance of its indicators. The AVE values of all seven variables ranged from 0.50 to 0.71, the rule of thumb for convergent validity is that the AVE values of each construct should be > 0.50. Our result indicated good convergent validity of all seven variables (see Table 6).

**Table 6:** Convergent validity

Latent Variables	The average variance extracted (AVE)	(Square rout of AVE/Indicators)
SR	**0.568**	*0.92*
IS	**0.67**	*0.981*
HLT	**0.61**	*0.96*
SW	**0.71**	*0.93*
SC	**0.5**	*0.972*
ES	**0.61**	*0.92*
SFT	**0.55**	*0.921*

Table 6 the diagonal elements in boldface are the average variance extracted (AVE) for each construct. These values should be greater than 0.50, which is rule of thumb to evaluate convergent validity. The diagonal elements in italics faces are square roots of average variance extracted (AVE) for each construct will be compared with intra-constructs correlations to examine the discriminatory validity.

#### Discriminatory validity

Discriminant validity explain the deviation among the seven latent variables in the factor analysis. To check the discriminatory validity, we calculated the square root of AVE and divided it by the total number of indicators in each variable. The discriminatory values ranged from 0.92 to 0.98 for seven variables. The discriminatory validity of the scale was compared with the intra-construct correlation values. The discriminatory values of each variable is greater than the value of the intra-constructs correlation, which showed appropriate discriminatory validity in this study (see Table 7).

**Table 7:** Discriminant validity

	SR	LIS	HLT	SW	SC	ES	SFT
SR	**0.92**						
LIS	0.490	**0.98**					
HLT	0.52	0.47	**0.96**				
SW	0.67	0.40	0.86	**0.934**			
SC	0.38	0.40	0.35	0.38	**0.971**		
ES	0.67	0.55	0.41	0.50	0.67	**0.922**	
SFT	0.39	0.41	0.47	0.48	0.38		**0.922**

The diagonal elements in boldface are the square root of the average variance extracted (AVE). This value should be greater than intra-construct correlations (off-diagonal elements) for adequate discriminatory validity

#### Criterion validity

It is important to compare the scores obtained from the new scale with the scores of the already validated measure that is considered the criterion for the current scale. To establish criterion validity, Pearson correlation coefficients of scores between the active aging scale and the successful aging scale showed in Table 8. Scales correlation was high between the same or similar domains than between non-similar domains. For example, the healthy domain items were strongly correlated with the domain of healthy lifestyle (0.05), self-reliance scale was correlated with adaptive coping(*r* =  > 0.40) Three out of seven hypothetical scales showed strong correlations(*r* = 0.40) with significance (*P*. < 0.002) in comparison. Self-reliance was strongly correlated with adaptive coping(*r* = 0.46), engagement with life (*r* = 0.48), healthy lifestyle with healthy (*r* = 0.5.6), engagement(*r* = 0.44), and adaptive coping(*r* = 0.54). The inter-scale correlation between AA-PAK 34 and SAS-20 instrument correlation was high between similar domains as compared to different domains presented in Table 8. For example, the healthy lifestyle, healthy, learning, and integration into society were strongly correlated with engagement with life, and adoptive coping in a successful scale with a coefficient (> 0.5).

**Table 8:** Correlation coefficient between AA-PAK and SAS

	Healthy lifestyle	adaptive coping	Engagement with life
Self-reliance	0.32*	0.46**	0.48**
learning and integration	0.42**	0.48**	0.42**
healthy lifestyle	0.56**	0.54**	0.44**
spiritual wisdom	-0.21	0.42**	0.54**
social contribution	0.**52**	0.112*	0.41**
economic security	0.023	0.131**	0.285**
Strengthen family ties	0.31**	0.-131	0.13*

^*^ = *P*. < 0.05, ** = *P*. < 0.02, *** = *P*. < 0.001

#### Structured validity of the scale

**Fig. 2:**
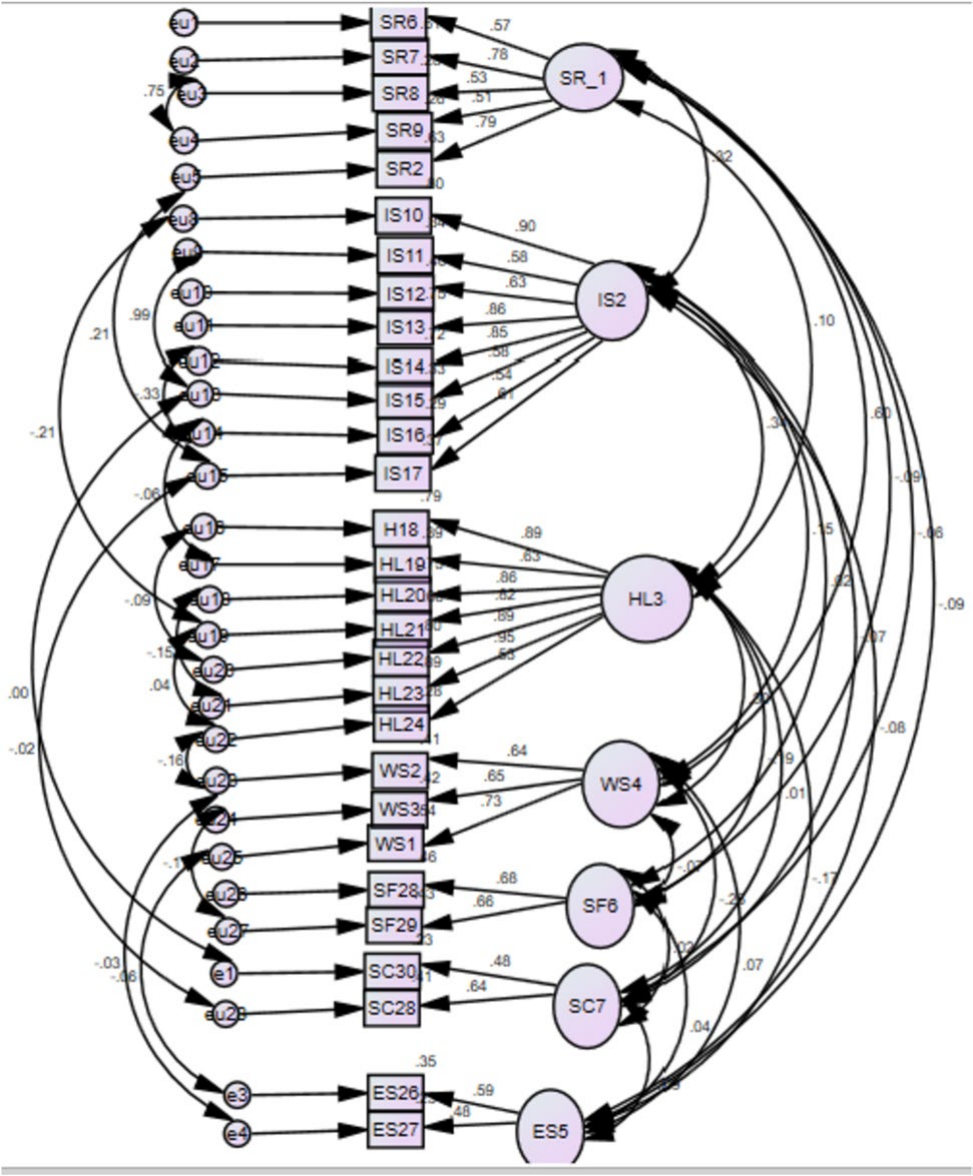
Unstandarized regression rate in item loading analysis model in the default model. Note:Aabbreviations;SR = selfreliance,IS = integrateinto society,HL = healthy life,WS = Spritual wisdom,SF = stregthen family ties, SC = social contribution,ES = economic security

**Fig. 3:**
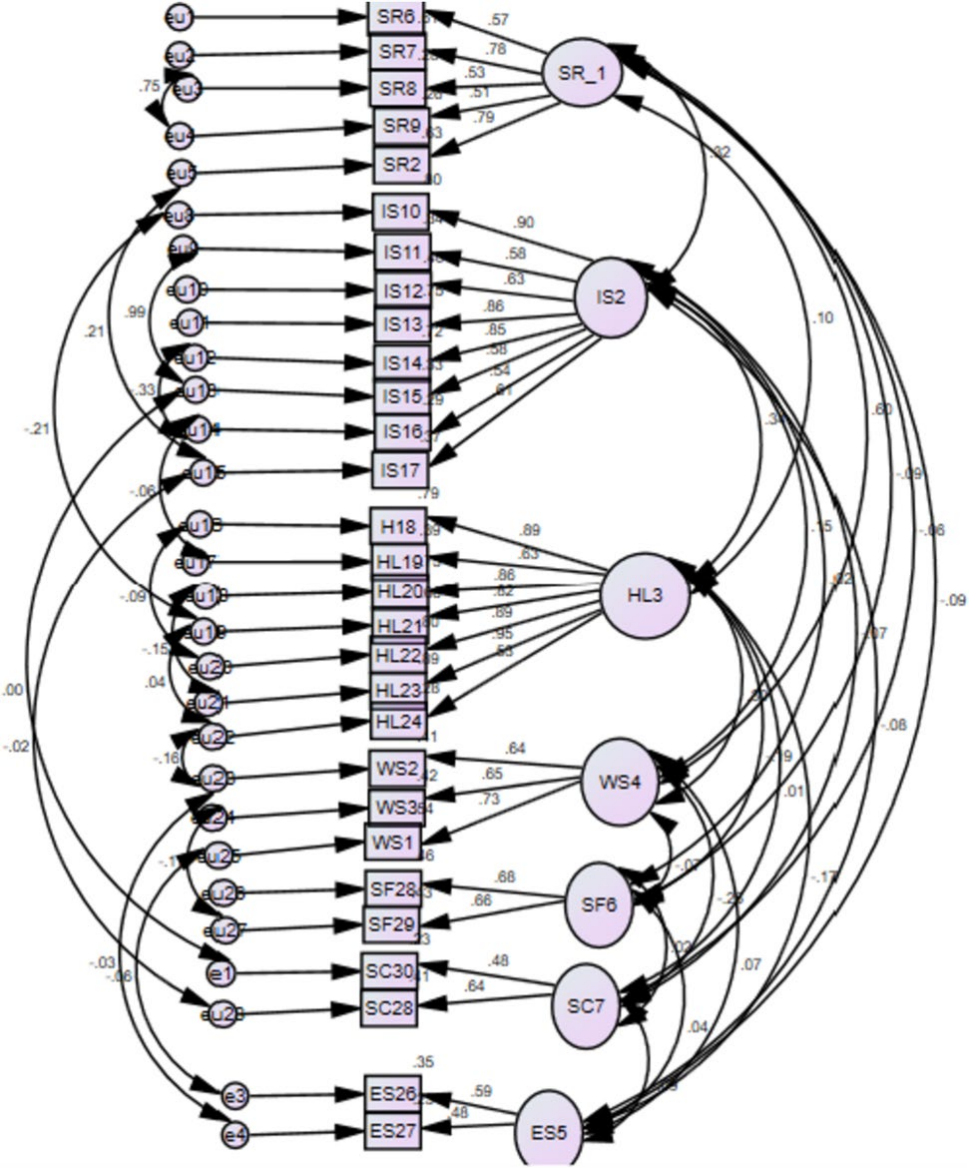
Standarized regression rate in item loading in the factor analysis model

For the structural validity of instruments, several fit indices were estimated to see the overall fit model for the measures through structural equation modeling. The seven factors solution matches well according to the CFA results for the scale's 34-item structure. In the confirmatory factor analysis, Chi-square X^2^ /df is 418.18, (df.2.2) in the standardized model which is < 3 indicated a good fit of the model. The value of RMSEA (0.12), GFI0.64, and CFI (0.68) was not significant in the default model, which was managed through error variance in the standardized model. The RMSEA is 0.042 which is < 0.80 indicating acceptable to model fit, the goodness of fit index(GFI) value is 0.92 which is > 0.90 is acceptable, the adjusted goodness of fit index(AGFI) value is 0.94 showing the good fit of the model, comparative fit index(CFI) values are 0.92 to 0.96 respectively. The CFI value is 0.92 as > 0.80 is acceptable. Figures 2, 3 and Table 9 shows model fit structure of the scale.

**Table 9:** Model fit in confirmatory factor analysis of 34 items of AA-Pak

Active Aging Scale	Model	X^2^(df)	NFI	IFI	TLI	CFI	RMSEA ( df)	$\nabla {x}^{2-}$($\Delta {x}^{2}$ df)
	Unstandardized Model	493. 23(52)	0.68	0.72	64.56	0.68	0.12	
	Standardized model		0.88	0.89	0.92(> 0.90)	0.91(> 0.80)	0.64 (< 0.80)	418.18(28)

*Abbreviations*: *IFI* Incremental Fit Index, *NFI* Normed Fit Index, *TLI* Tucker-Lewis Index, *CFI* Comparative Fit Index, *RMSE* Root Mean Square Error of Approximation, and *χ* ^*2*^ Chi-Square correlation coefficient

1. Model.1 is the default model without adding error variance

2. Mmodel.2 is the standardized after adding error variances

Note1. Chi-square test: The value is a statistical test of the goodness of fit of a factor model, prepare to observe covariance matrix with a proposed covariance matrix.

Note2. Root Mean Squared Error of Approximation (RMSEA): RMSEA is a measurement of the estimated disagreement between the population and the population in model-implied the covariance matrices per degree of freedom.

Note3. Tucker Lewis Index (TLI): TLI based on the concept of contrasting the proposed factor model to a model in which absolutely no assumptions made regarding the interrelationships between any of the elements.

Note4. Comparative Fit Index (CFI): The incremental relative fit index, or CFI, evaluates how much better a researcher's model fits than a baseline model.

#### Model with correlated errors

Error of covariance in factor analysis research is significant to hold items without deletion [31], in order to achieve a satisfactory fit, we reanalyzed the data set while leaving error covariance in the model. The estimations were high in three factors between items and factors, which are high in CFA. The active aging scale’s seven-factor structure and composite scores have both been studied. Model visual representation showing factor loading and model fit. Figure 2 depicted CFA model fit structures of the scale. The dimensionality of the model was assessed. Figure 3 has confirmed that the dimensionality of the item variance was significant in EFA as the standardized regression rates show values above 0.40 in the regression equilibria with standard errors. The error variance show that there is high variability among factors and it indicated that the seven factors are impartially related to each other in the model.

#### Test–retest reliability

To assess the stability of the last version of the AAS-Pak with 34 items, we re-approached 35 older adults who live in the Peshawar district, who showed a mean score of 98.2 at time one, and a mean score of 95.60 at time two. The two groups scores' Pearson correlation coefficient, which measures the stability of the scale with Cronbach Alpha values of 0.92 and 0.88, respectively. Additionally, the seven subscales' correlations between time one and time two varied from 0.77 to 0.90 (see Table 10). The 34-item AAS-Pak is stable in terms of test–retest reliability, according to the findings Table 4. Test–retest stability check of AAS-Pak with sub-sample.

**Table 10:** Test–retest reliability of the AAS-Pak 34, *N* = 35

First time data			Data after 2 weeks			*r* =
	Mean	Std. Deviation		Mean	Std. Deviation	
SR	16.6188	4.36808	SR	22.319	5.017	0.84
LIS	27.1688	6.83335	IS	31.625	9.612	0.78
HLT	23.0125	4.79122	HL	20.025	5.049	0.84
SW	10.1125	2.92675	SWL	15.425	2.762	0.77
ES	10.5500	2.16838	ES	9.7813	1.922	0.81
SFT	7.4000	2.22677	FS	12.45	1.722	0.85
SC	6.6375	1.51507	SC	6.5563	1.999	0.79
TOTAL	98.2125	11.95978	TOTAL	95.60.113	10.74	0.8
Cronbach Alpha = 0.92				Cronbach Alpha = 0.88		

In the Table 10, we calculate how repeatable the participant's performance is, i.e., how stable their scores are over time is showing consistency beteeen two-time data collected from the participants with Cronbach’s Alpha 0.92, and 0.88 in the second data.

## Discussion

In light of the limited availability of research tools in the local “Urdu” language for assessing active aging levels in Pakistani older adults, the primary goal of this study was to address this challenge by focusing on the translation, and validation of the Active Aging Scale, which consists of 36 items. Another purpose was to determine its acceptability, suitability, and applicability in the specific context of Pakistan.

The translation process is split into two halves in order to accomplish this purpose. Using expert panel discussion, content validity check, and a preliminary pilot study with 30 participants, Sect. 1 of the study focused on the translation of the Active Ageing Scale and looked at its temporal validity and reliability. The outcomes of this stage showed that the scale was stable and suitable for further evaluation, with a Cronbach’s Alpha of 0.80, CVI, and SCVI > 0.90. Two items were removed from the scale based on item variance value < 0.30, securing 34 items for further psychometric tests. According to prior studies, the item reduction was anticipated during the initial phase of the scale translation process.

In part two, the psychometric tests of the Urdu-version scale consisted of 34 items that measured the reliability, and validity of the scale. Data were collected from (*n* = 160) both, men and women made up an equal number of the sample. Data obtained from the participants were analyzed for internal consistency, as Cronbach Alpha was α = 0.88, which is considered an excellent internal consistency. Factor analysis (exploratory and confirmatory factor analysis) sustained the seven components with 34 items such as self-reliance, learning, and integration into society, healthy lifestyle, spiritual wisdom, social contribution, financial security, and strengthened family ties in the model. The results revealed that the scale has sustained the structure of the original scale with the seven components. In the literature different concepts were generated in terms of social contribution, healthy lifestyle, independent self-care, mental wellbeing, participation in social affairs, and autonomy were measured as active aging components [34]. This study claimed that item pooling was created at a point covering the topics available in the original scale and many others in different contexts [20].

Psychometric analysis in section two involved the establishment of construct validity using confirmatory factor analysis on a group of older adults (*n* = 160). When we looked at the descriptive information of the older adults, the proportion of uneducated participants was quite high, at 58% of the total, but the participants communicated in the Urdu language. The age distribution of the participants is over 60 to 70 years old (62%). The fact that the majority of the older adults live in a joint family system shows a strong family support system in Pakistan. The financial dependency on other family members was high, which is consistent with the previous study in Thailand [14], but not consistent with a European study in which older adults were not dependent financially on their children [20]. In our study, female participants had a higher dependency ratio on their children than male participants, which is not surprising because females are forced to stay at home and take sole responsibility for domestic affairs and look after their children which is linked with the value system of the Muslim community [1]. After examining the previous studies, it has been noticed that huge different in terms of demographic values conducted for active aging in other studies [19, 28, 35].

In the factor analysis, 34 items were loaded clearly; five items for self-reliance, eight items for learning and integration into society, seven items for healthy behavior, three items for spiritual wisdom, two factors for economic security, two for social contribution, and two were for strengthen family ties. In the construct validity, the scale maintained its seven factors structure as mentioned in previous studies in different languages [29] but the items loading of three factors were not similar to the original scale. This variance can be due to the development of culturally sensitive scales in different countries with some variations in the components that may influenced.

The seven factors were correlated with each other supporting the entire scale as a valid and good fit with a significance of (*P*.0.01),RMSEA value of 0.42, (GFI) value is 0.92 which is > 0.90 is acceptable in this model. According to Fig. 2, the active aging scale's factor loading spans from 0.42 to 0.90. These results showed that the translated instrument obtained 34 items with seven factors evaluated in the current research equally applicable, and valid to Pakistani culture, as were applied in the native culture [14]. The items vary in each factor such as self-reliance consisting of five items, one eliminated due < 0.3 scores in item variance, focused on self-care, tasks related to self and family care, and performing various activities in the household. From the perspective of older adults, being able to do what they wish is meaningful for their autonomy and implies that they can manage their lives on their own [19]. In the collectivistic nature of Islam and cultural norms, the people were interconnected, and they were held accountable for one another [34]. This notion was consistent with the valued concept of individualism among older adults Western people [36], and Yorozuya’s study in Japan [23]. The concurrent validity of the scale was checked comparing by obtained data from active aging scales and the Successful Aging Scale, which was translated in the same language. A significant positive relationship between AAS-Pak and SAS with the SR = 0.85**, LIS = 0.75**, HLT = 0.81**, and the overall scale value (*r* = 0.86**, with significant value of (*P* =  < 0.001). Surprisingly, our study results were consistent with the results of other studies conducted in another context, for example, health related questionnaires used in Japan [23], and the successful aging scale used in Turkey study to check the criterion validity of the active aging scale [28]. The concept of active aging is very diverse therefore, active aging scales were gone through items addition, and deletion based on the context in the previous studies. Therefore, there is a lack of a standardized active aging scale. According to the WHO policy framework, the active aging level can be assessed effectively if the scale used according to the demographic characteristics, and needs of the older adults within a specific area [17, 37].

The AA-PAK claimed to be a valid and reliable scale to assess the WHO proposed seven active aging determinants. The overall AAS-Pak internal consistency of seven variables was consistant as the average Cronbach’s Alpha value was 0.88 in this study. According to Zamanzadeh [30] a scale is considered to be extremely reliable if its alpha coefficient is 0.80 or higher. The test–retest reliability of the scale is showing mean scores of 105 the first time with Cronbach’s Alpha 0.92 and a mean score of 106.80 the second time with Cronbach’s Alpha 0.88 showing the stability of the scale. The scale is seen to be stable and valid in various contexts with some attrition and deletion of the items as per their relevancy to the context [15, 19, 23]. These results demonstrated that the AAS-Pak is a valid instrument to assess the level of active aging in community-based older adults in Pakistan. The 34 items were culturally acceptable, all items were consistent with the other studies with different cultural backgrounds [22]. The active aging scales were translated into different languages. A systematic review of 20 included studies revealed that there are multiple active aging scales available based on the context, however, all the active aging scales maintained an average of five dimensions of active aging, the same study recommends further psychometric testing of an active aging scale to use in different context [18]. It is evident that the scholars in the previous studies used different active aging scales with different names [18]. According to Baoting the social beliefs, emotions, and needs of older adults may not be different from context to context, however, some traditional values may compromise the scale validity if applied in a different contexts [19, 31].

### Limitations of the study

The scale is exclusively adaptive and its translation into Urdu language may not address all aspects of active aging, as this is a complex concept. Applying the scale to individuals with lower education levels in a specific region may not accurately represent the broader society in other regions where the quality of life could be high. The participants' low literacy could pose challenges to their understanding; can potentially compromise the scale's reliability. For future studies, it may be necessary to include additional items obtained through in-depth exploration of older adult’s individuals' perspectives on active aging through conducting a need assessment study. Comparing these findings with an active aging scale developed in a different cultural context would be valuable in determining the unique and universal dimensions of the Active Aging-Pak.

## Conclusion

The Active Aging Scale-Pak might be Pakistan's first multidimensional active aging scale that is relevant, valid, and culturally contextualized. The 34-item Active Aging-Pak is culturally sensitive, valid, and reliable study tool that can be used to assess active aging in older adults living in Pakistan, indicating that it may be applied in both community and clinical practice settings.

## Data Availability

All the data is secured and available from the corresponding authors. The corresponding authors will provide all the data on the demand of the journal.

## Abbreviations

AA-PAK: Active aging scale for Pakistan
WHO: World Health organization
AS: Successful Aging Scale
ISPOR: International Society of Pharmacy Economic and Outcome Research
DHO: District Health Office
IRB: Institutional Review Board
AAI: Active Aging Index
ZZU: Zhengzhou University
CVI: Content validity index
CFA: Confirmatory factor analysis
IFI: Incremental Fit Index
TLI: Tucker Lewis Index
RMSEA: Rout mean square error of approximation
AVE: Average variance extracted
